# Pelvic Lymph Node Dissection With Polymer Ligation Clips Significantly Reduces Hospital Stay Compared to Vessel Sealer: A Retrospective Study

**DOI:** 10.7759/cureus.73958

**Published:** 2024-11-18

**Authors:** Hirotaka Yokoyama, Toru Sugihara, Hiroto Kishino, Atsushi Yanase, Risako Watanabe, Kaori Endo, Jun Kamei, Ei-ichiro Takaoka, Satoshi Ando, Tetsuya Fujimura

**Affiliations:** 1 Urology, Haga Red Cross Hospital, Moka, JPN; 2 Urology, Jichi Medical University, Shimotsuke, JPN; 3 Urology, The Fraternity Memorial Hospital, Sumida, JPN; 4 Urology, The University of Tokyo Hospital, Bunkyo, JPN

**Keywords:** pelvic lymph node dissection, pelvic lymphocele, polymer ligation clip, robot-assisted radical prostatectomy, vessel sealer

## Abstract

Introduction

Lymphocele is a typical complication of pelvic lymph node dissection (PLND) in robot-assisted radical prostatectomy (RARP). This study aimed to compare postoperative lymphatic leakage between the polymer ligation clip and vessel sealer, and evaluated the costs associated with the former.

Methods

The study enrolled patients who underwent RARP with PLND at our institution between April 2018 and March 2023 and were treated with a vessel sealer (LigaSure Blunt Tip 44 NC^TM^; Medtronic, Dublin, Ireland) until September 2021, and Hem-o-lok polymer ligation clips^TM ^(Teleflex, Wayne, PA, USA) thereafter. The drain was removed on the third postoperative day or later, when the daily drainage volume was less than 200 mL.

Results

A total of 81 patients underwent RARP with PLND, which resulted in a significant increase in the operative (p = 0.016) and lymph node dissection times (p = 0.008) in the clip group. The total drainage volume (p = 0.048) was smaller, and the drain removal time (p = 0.039) and postoperative hospital stay (p < 0.001) were shorter in the clip group. Moreover, the device costs for the clips were lower than those for the vessel sealer.

Conclusion

Polymer ligation clips are associated with improved postoperative lymphatic leakage, shorter length of hospitalization, and lower costs, compared with vessel sealers.

## Introduction

Pelvic lymph node dissection (PLND) is an effective method for detecting lymph node metastases in patients with prostate cancer undergoing radical prostatectomy, and appropriate staging can aid in the selection of adjuvant or salvage therapy [1]. However, pelvic lymphocele is a typical complication of PLND [1]. Previous studies have evaluated the incidence and risk factors for pelvic lymphocele after PLND [2,3]. Several methods have been employed for PLND, including bipolar electrocautery, surgical clips, and vessel sealers [4].

Hem-o-lok clips^TM ^(Teleflex, Wayne, PA, USA), which are commonly used during surgery, can be used to ligate tissue without heat, maintain ligature force, and can be removed and reattached easily, as necessary. The clip is composed of an inert, non-conductive, radiolucent, and non-absorbable polymer, which does not interfere with computed tomography (CT), magnetic resonance imaging, or X-ray. LigaSure^TM ^(Medtronic, Dublin, Ireland) was first used clinically in 1998 and has since been employed in numerous surgeries [5]. This system functions by denaturing collagen and elastin, fusing and integrating tissue walls, thereby creating a seal. It can seal up to 7 mm of blood vessels, lymphatic vessels, and tissue bundles, with a lateral heat dissipation of less than 2 mm.

Furthermore, a study indicated that the application of LigaSure reduced the total operative time and the requirement for blood transfusion, compared with the conventional ligation method [5]. Despite the availability of various PLND methods, lymphoceles develop after robot-assisted radical prostatectomy (RARP) with PLND in approximately 60% of cases [6]. Increased postoperative lymph leakage can delay the removal of the intraperitoneal drain and prolong postoperative hospital stay. To the best of our knowledge, no study has explored the significance of vessel sealers and clips in intraperitoneal drain removal. In this study, we compared and evaluated the drainage volume, treatment outcomes, and surgical costs of RARP with PLND using polymer ligation clips and vessel sealers at Jichi Medical University Hospital.

## Materials and methods

We retrospectively analyzed the data of patients who underwent RARP with PLND for prostate cancer at Jichi Medical University Hospital between April 2018 and March 2023. PLND was indicated for patients with Gleason score pattern 5 detected on prostate biopsy, or prostate-specific antigen (PSA) levels >20 ng/mL at diagnosis. The PLND areas were set in accordance with extended PLND delineated by the National Comprehensive Cancer Network (NCCN) guidelines [7]. Extended PLND entails the removal of nodes overlying the external iliac artery and vein, nodes within the obturator fossa located cranially and caudally to the obturator nerve, and nodes medial and lateral to the internal iliac artery [7]. According to the NCCN classification, extended PLND provides the most accurate information for staging and prognosis [7]. A vessel sealer (LigaSure Blunt Tip 44 NC) was used between April 2018 and September 2021, while polymer ligation clips (Hem-o-lok clip applier) were employed thereafter, between October 2021 and March 2023. Patients in whom a vessel sealer was used were designated as the vessel sealer group, whereas those in whom Hem-o-lok clips were used were designated as the clip group. PLND was performed with the conventional technique using robotic electrocautery scissors and fenestrated bipolar instruments. Furthermore, Hem-o-lok clips were placed at the proximal and distal boundaries of PLND. The vessel sealer was used at similar proximal and distal boundaries but without the use of Hem-o-lok clips. The drain was removed on the third postoperative day or later, contingent on a daily drainage volume of <200 mL. Perioperative complications were evaluated at one and three months postoperatively using the Clavien-Dindo classification [8]. We evaluated and compared the drainage volume, surgical time, bleeding volume, perioperative complications, pathological results, and surgical costs for RARP with PLND between the clip and vessel sealer groups.

Statistical analysis was performed using the Mann-Whitney U test for continuous variables and Fisher’s exact test for nominal variables. Statistical significance was set at a p-value of <0.05. Since the Mann-Whitney U test is being used, there is no t-value in the statistical analysis. All statistical analyses were performed using EZR software (Saitama Medical Center, Jichi Medical University, Saitama, Japan) [9].

## Results

**Figure 1:**
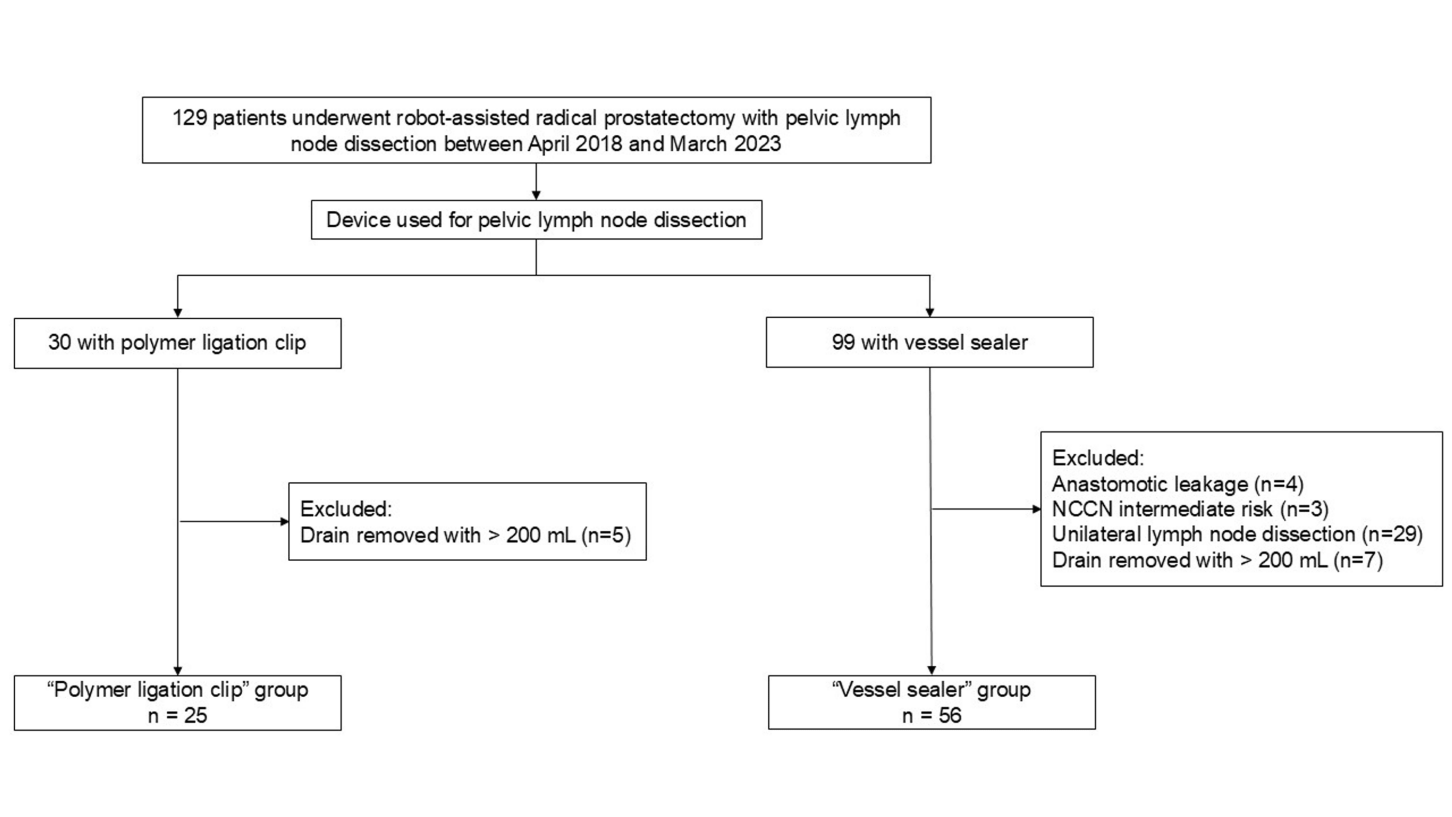
Patient selection flow chart NCCN: National Comprehensive Cancer Network

During the study period, 129 patients with prostate cancer underwent RARP with PLND. Polymer ligation clips and vessel sealers were used in 30 and 99 patients, respectively. We analyzed the data of 25 patients in the clip group and 56 patients in the vessel sealer group, after excluding those who did not meet the inclusion criteria, such as patients with postoperative urinary leakage (Figure 1).

The patients’ baseline demographic characteristics are shown in Table 1. Age, body size, comorbidities, and tumor stage did not differ significantly between the two groups.

**Table 1:** Baseline demographic characteristics of patients who underwent pelvic lymph node dissection during robot-assisted radical prostatectomy using polymer ligation clips or vessel sealers PSA: prostate-specific antigen

	“Polymer ligation clip” group, n = 25 (median (range) or n (%))	“Vessel sealer” group, n = 56 (median (range) or n (%))	p-value
Age, years	72 (57-79)	69.5 (52-79)	0.126
Body mass index, kg/m^2^	24.5 (17.7-28.4)	23.8 (18.8-31.7)	0.915
Charlson comorbidity index			0.08
0	20 (80)	39 (69.6)	-
1	1 (4.0)	10 (17.9)	
2	4 (16)	3 (5.4)	
3+	-	4 (7.1)	
Peripheral vascular disease	1 (3.3)	3 (5.4)	0.722
Myocardial infarction	-	4 (7.1)	-
Diabetes mellitus	3 (12)	10 (17.9)	
Solid tumor	1 (4.0)	3 (5.4)	
Connective tissue disease	1 (4.0)	1 (1.8)	
PSA at diagnosis, ng/mL	20 (4.96-72)	9.83 (4.47-74)	0.431
Clinical T stage			0.926
≤cT2c	21 (84)	46 (82.1)	-
cT3a	3 (12)	7 (12.5)	
cT3b	1 (4.0)	2 (3.6)	
cT4	-	1 (1.8)	
Gleason score			0.698
≤7	2 (8.0)	5 (8.9)	-
8	6 (24)	9 (16.1)	
9≤	17 (68)	42 (75)	
Prostate volume, cm^3^	34.1 (13.6-65)	31.8 (15-89)	0.69

The surgical outcomes are presented in Table 2. The median operative and PLND times were shorter in the vessel sealer group than those in the clip group (p = 0.016 and p = 0.008, respectively). Significant differences in the positive resection margins or pathological findings were not observed between the two groups. Complications within 90 days of surgery included symptomatic lymphocele, which occurred in one patient in the clip group and required puncture drainage and antibiotic therapy. There was no significant difference in the overall postoperative complications between the two groups (p = 0.720).

**Table 2:** Surgical outcomes in patients who underwent pelvic lymph node dissection during robot-assisted radical prostatectomy using polymer ligation clips or vessel sealers

	“Polymer ligation clip” group, n = 25 (median (range) or n (%))	“Vessel sealer” group, n = 56 (median (range) or n (%))	p-value
Operative time, min	250 (150-367)	219 (119-398)	0.016
Console time, min	196 (109-301)	171 (86-363)	0.054
Pelvic lymph node dissection time, min	52 (13-89)	35 (14-73)	0.008
Bleeding, mL	150 (5-700)	200 (5-800)	0.291
Prostate volume, gr	42 (22.5-70)	35.5 (4-123.5)	0.213
Pathological T stage			0.077
pT2	7 (28)	25 (44.6)	-
pT3a	11 (44)	10 (17.9)	
pT3b	7 (28)	18 (32.1)	
pT4	-	1 (1.8)	
Pathological N stage			0.162
pN1	1 (4.0)	9 (16.1)	-
Dissected lymph nodes, n	9 (3-39)	13 (4-27)	0.482
Positive lymph nodes, n	0 (0-1)	0 (0-2)	0.13
Gleason score			0.346
≤7	6 (24)	22 (39.3)	-
8	4 (16)	6 (10.7)	
9≤	15 (60)	25 (44.6)	
Positive resection margin	15 (60)	21 (37.5)	0.235
Postoperative complications - Clavien-Dindo classification			0.72
Grade I	15 (60)	41 (73)	-
Grade II	1 (4.0)	7 (12.5)	
Grade IIIa	1 (4.0)	2 (3.6)	
Symptomatic lymphocele	1 (4.0)	-	

Table 3 presents the postoperative drain effluent outcomes of both groups. The median total drainage volume was significantly lower in the clip group, at 235 mL, compared to 540 mL in the vessel sealer group (p = 0.048). Moreover, the duration of postoperative drain placement (p = 0.039) and postoperative hospital stay (p < 0.001) were significantly shorter in the clip group.

**Table 3:** Postoperative drainage effluent outcomes in patients in whom polymer ligation clips or vessel sealers were used for pelvic lymph node dissection POD: postoperative day

	“Polymer ligation clip” group, n = 25 (median (range) or n (%))	“Vessel sealer” group, n = 56 (median (range) or n (%))	p-value
Postoperative drain removal, POD	4 (3-8)	4 (3-13)	0.039
Drain effluent volume, mL			
POD1, n = 25 vs. 56	100 (27-330)	110 (10-380)	0.396
POD2, n = 25 vs. 56	100 (2-495)	137 (26-414)	0.487
POD3, n = 15 vs. 39	79 (20-472)	134 (7-643)	0.148
POD4, n = 3 vs. 25	190 (92-255)	140 (10-524)	0.698
POD5, n = 2 vs. 15	185 (170-200)	181.5 (25-490)	0.819
POD6, n = 1 vs. 10	305 (305-305)	223.5 (35-607)	0.462
POD7, n = 1 vs. 5	35 (35-35)	155 (96-678)	0.333
Total	235 (34-1708)	540 (52-4655)	0.048
Postoperative length of hospital stay, day	7 (7-10)	8 (5-22)	<0.001

Table 4 presents the data on surgical expenses. In the clip group, 22 polymeric ligature clips were used intraoperatively, and the median cost per operation was JPY 23,760. Only one unit was used during surgery in the vessel sealer group, and the cost was JPY 94,760. The device cost per surgery was significantly lower in the clip group than that in the vessel sealer group (p < 0.001).

**Table 4:** Device cost comparison in 25 patients treated with polymer ligation clips and 56 patients treated with vessel sealers for pelvic lymph node dissection during robot-assisted radical prostatectomy JPY: Japanese yen

	“Polymer ligation clip” group, n = 25 (median (range) or n (%))	“Vessel sealer” group, n = 56 (median (range) or n (%))	p-value
Number of units used	22 (10-38)	1 (1-1)	<0.001
Cost per operation, JPY	23,760 (10,800-41,040)	94,760	<0.001

## Discussion

We compared the postoperative outcomes and device-related costs of polymer ligation clips with those of vessel sealers for PLND in patients who underwent RARP. We found that polymer ligation clips were associated with improved postoperative lymphatic leakage, shorter hospital stays, and lower costs than vessel sealers.

A systematic review reported that, although radical prostatectomy followed by PLND does not improve survival or oncological outcomes, it remains an effective method for detecting lymph node metastases [10]. When determining the indications for PLND, the benefits must be weighed against the complications and costs. In our hospital, PLND is indicated for patients with Gleason score pattern 5 detected on prostate biopsy or PSA levels >20 ng/mL at diagnosis, who are stratified into the high-risk or very high-risk groups by the NCCN classification. Recently, a preoperative nomogram was devised to stratify the risk of lymph node invasion based on prostate cancer characteristics and select the indications for PLND [11]. Furthermore, the European Association of Urology guidelines state that PLND may be omitted in cases where the risk of lymph node metastasis on the nomogram is <7% [12,13]. Similarly, the NCCN guidelines suggest the omission of PLND in cases where the risk of lymph node metastasis is <2%, as indicated by the nomogram [7]. Regarding our cases, the N1 probability predicted by the Briganti nomogram [12] ranged from 13% to 100%, and there were no cases with a probability of less than 7%.

A higher number of lymph node-positive cases were observed in the vessel sealer group (4.0% vs. 16.1%, p = 0.162). Because there is no significant difference in the number of lymph nodes removed, this trend would be coincidental; however, we would like to continue to examine this trend in the future as we accumulate more cases.

The typical intraoperative complications associated with PLND include damage to the major blood vessels, ureter, and obturator nerves, whereas pelvic lymphocele, lymphedema, and deep vein thrombosis are among the archetypal postoperative complications [6]. The incidence of symptomatic lymphocele reportedly ranges from 0% to 10% [1,6], and we encountered only one such case in this study (1%). The incidence of Clavien-Dindo grade 3-4 complications related to PLND reportedly ranges from 0% to 5% [1], and three such cases (3.7%) were encountered in our hospital, akin to the reports from other hospitals.

A previous study compared the outcomes of patients treated with LigaSure and Hem-o-lok clips during RARP [14]. Overall, the data from 375 cases (LigaSure, n = 125; Hem-o-lok clip, n = 250) were analyzed using propensity score matching. Similar to our study, the median operative and console times were significantly shorter in the LigaSure group than in the Hem-o-lok clip group (p < 0.001 and p = 0.003, respectively). The rate of positive resection margins was significantly lower in the LigaSure group than in the clip group (p = 0.002). We used LigaSure only for PLND, and clips were used in all cases to avoid thermal injury in all procedures other than PLND, including treatment of the lateral pedicle. Therefore, in our study, the rate of positive resection margins was not included in the comparison between LigaSure and the clips. Moreover, as the analysis excluded nerve-sparing cases, further studies are needed to improve urinary abstinence and erectile function in nerve-sparing cases using LigaSure and clips, whose postprocedural recovery remains debatable. The average cost of using the clip was JPY 1,308,278, which is significantly lower than that of LigaSure (JPY 1,705,889). Consistent with this study, the use of a clip reduces costs.

New vessel sealers have recently become available. A prospective study enrolling 114 patients reported the results of a comparison of the incidence of pelvic lymphoceles in RARP using robotic vessel sealers and polymer ligation clips [15]. PLND was performed on one side using a robotic vessel sealer and clips on the other side in the same patient. Lymphoceles were evaluated using an abdominal CT performed three months after surgery and defined as a cavity of 3 cm or more in diameter. The robotic vessel sealer was used to dissect 3.1 lymph nodes, and clips were used to dissect 3.3 nodes, with no significant difference (p = 0.35). The mean unilateral PLND times were 11.3 and 11.1 minutes for the robotic vessel sealer and clips, respectively; the difference was not significant (p = 0.62). No significant differences were observed in the number of lymphatic cysts between the 10 cases treated with robotic vessel sealers and the 12 cases treated with clips (p = 0.41). Symptomatic lymphocele was not observed in either group. Although the incidence of lymphocele did not differ significantly between the robotic vessel sealer and clips, the expense of the robotic vessel sealer is a concern, as it costs JPY 89,250 per case, similar to other vessel sealers.

Clips have been reported to prolong the operative time and increase the risk of vascular and intestinal injury compared with vessel sealers. However, the cost-effectiveness, reduction in total drainage volume, reduction in the duration of drain placement (i.e., the number of days in which the drain can be removed), and length of hospital stay may be beneficial. This study suggests that polymer ligation clips reduce surgical costs, decrease the total drainage volume, and shorten the duration of drain placement and hospital stay compared with vessel sealers. However, our study has several limitations, including its small sample size, the lack of long-term follow-up data, retrospective study design, the absence of randomization, the possibility of a potentially large drainage volume in patients with postoperative urinary leakage, and the exclusion of patients in whom the drain was removed at a volume of ≥200 mL. Furthermore, since the creatinine level of the drainage fluid was not measured, cases with potential urinary leakage from the ureterovesical anastomosis might have been included. The price of the product and the number of clips used in each hospital would result in differences in the possible cost savings. These factors limit the generalizability of the findings; further investigation is needed in future research.

## Conclusions

We observed a low incidence of typical intraoperative complications associated with PLND, with only one case of symptomatic lymphocele and three cases of Clavien-Dindo grade 3-4 complications. While clips may prolong the operative time and increase the risk of vascular and intestinal injury compared with vessel sealers, they offer potential benefits in cost-effectiveness, reduced drainage, shorter drain placement time, and decreased hospital stay. Our study suggests that polymer ligation clips reduce surgical costs, decrease the total drainage volume, and shorten the duration of drain placement and hospital stay compared with vessel sealers for PLND during RARP.
